# Floquet prethermalization and Rabi oscillations in optically excited Hubbard clusters

**DOI:** 10.1038/s41598-021-97104-x

**Published:** 2021-09-09

**Authors:** Junichi Okamoto, Francesco Peronaci

**Affiliations:** 1 Institute of Physics, University of Freiburg, Hermann-Herder-Str. 3, 79104 Freiburg, Germany; 2 EUCOR Centre for Quantum Science and Quantum Computing, University of Freiburg, Hermann-Herder-Str. 3, 79104 Freiburg, Germany; 3 Max Planck Institute for the Physics of Complex Systems, Nöthnitzer Straße 38, 01187 Dresden, Germany

**Keywords:** Condensed-matter physics, Electronic properties and materials

## Abstract

We study the properties of Floquet prethermal states in two-dimensional Mott-insulating Hubbard clusters under continuous optical excitation. With exact-diagonalization simulations, we show that Floquet prethermal states emerge not only off resonance, but also for resonant excitation, provided a small field amplitude. In the resonant case, the long-lived quasi-stationary Floquet states are characterized by Rabi oscillations of observables such as double occupation and kinetic energy. At stronger fields, thermalization to infinite temperature is observed. We provide explanations to these results by means of time-dependent perturbation theory. The main findings are substantiated by a finite-size analysis.

## Introduction

Coherent optical manipulation of matter is a growing field of study due to the development of intense laser sources. Photoinduced phase transitions in many-body systems are now possible with non-thermal processes without loss of quantum nature^1–6^. For example, recent mid-infrared pump-probe experiments revealed exciting phenomena such as light-induced superconductivity^7–9^, ultrafast structural transitions^10,11^, and metastable charge ordering^12^, which are driven by short optical excitation of phonons. More direct manipulation of electronic states can be realized through the so-called Floquet engineering by continuous optical excitation^13–15^. Notable achievements in this direction are the prediction and realization of dynamical localization^16–18^ and topological band structures^19–22^.

In the high-frequency limit, Floquet theory provides a good description of low-energy phenomena in terms of effective static Hamiltonians. In this limit, heating is a rather minor effect even in interacting systems, since the drive frequency is away from any characteristic absorption energy of the system^23–25^. Thus, long-lived quasi-stationary states, which appear to be “thermalized”, are realized until the much later heating time scales^26,27^; such states are termed as Floquet prethermal states (FPSs). On the other hand, when the drive frequency is near an energy scale of a generic interacting system, or its submultiples, heating is expected due to possible photon absorption processes. In isolated systems, this leads to thermalization to infinite temperature^28^. Thus, resonant excitation makes the analysis by the Floquet picture more complicated.

As an alternative to the high-frequency limit, Floquet prethermalization is also observed in systems close to integrability, in which quasi-integrals of motion constrain the dynamics for finite but long times^29–42^. In particular, systems in the Mott-insulating phase of the infinite-dimensional Hubbard models were shown to display extremely long-lived prethermal states, even for frequencies close to resonance^36,41^. Further investigation of these long-lived quasi-steady states is of great importance to advance the Floquet engineering protocols for generic frequencies. To this end, we use a realistic finite lattice geometry and investigate the stability and controllability of possible FPSs.

In this work, we study Floquet prethermalization in two-dimensional (2D) Hubbard clusters under continuous optical excitation. The setup can be realized in solid-state systems such as quantum dot arrays under laser fields^43–46^ or in ultracold atoms in shaking optical lattices^26,27,47^. Starting from the Mott-insulating state, we calculate the driven time evolution by exact diagonalization. We find that Floquet prethermal states emerge even at frequencies resonant with absorption-peak energies, provided a small field amplitude. Remarkably, these long-lived quasi-steady states show Rabi oscillations of observables such as double occupation and kinetic energy. The spectral density shows that the system oscillates between the ground state and the one-photon excited state. For stronger excitation, the system goes to the infinite-temperature state, in general, and the spectral density is spread over many excited states. We elucidate the origin of the Rabi oscillations of the FPSs with the aid of time-dependent perturbation theory. A finite-size analysis indicates that the phenomena are robust for various lattice geometries up to fourteen sites, and possibly for larger systems.

## Formalism

**Figure 1:**
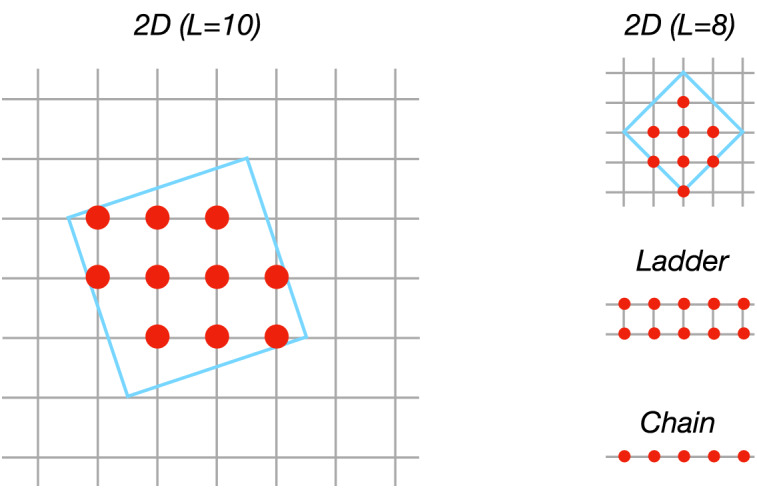
Two-dimensional cluster geometries. In this work, we consider mainly the cluster with $L=10$ sites with a periodic boundary (left), while other geometries (right) are also used for the finite-size analysis.

We study a half-filled Hubbard cluster in two dimensions under continuous optical excitation,

1 $$\begin{aligned} \begin{aligned} H(t)&= H_J (t) + U \sum _i n_{i\uparrow } n_{i\downarrow } ,\\ H_J(t)&= - \sum _{\mathinner {\langle {i,j}\rangle }, \sigma }J_0 e^{-i\frac{e}{\hbar }\int _{{\varvec{R}}_{j}}^{{\varvec{R}}_{i}} d{\varvec{r}} \cdot {\varvec{A}}(t)} c^{\dagger }_{i \sigma }c_{j\sigma } + \text {H.c.}\\&= - \sum _{\mathinner {\langle {i,j}\rangle }, \sigma }J_0 e^{-i \frac{e}{\hbar } {\varvec{l}}_{ij} \cdot {\varvec{A}}(t)} c^{\dagger }_{i\sigma }c_{j\sigma } + \text {H.c.}, \end{aligned} \end{aligned}$$

where $c_{i \sigma }$ and $c^{\dagger }_{i \sigma }$ are annihilation and creation operators at site *i* with spin $\sigma$, and ${\varvec{l}}_{ij} = {\varvec{R}}_{i} - {\varvec{R}}_{j}$ the bond vector connecting sites *i* and *j* at positions ${\varvec{R}}_i$ and ${\varvec{R}}_j$. Opposite spin densities $n_{i \sigma } = c^{\dagger }_{i\sigma } c_{i \sigma }$ are subject to a local Hubbard repulsion *U*. We take $\hbar = c = e = 1$, and use $J_0 = 1$ and $U = 6$, which ensures that the initial ground state is a Mott insulating state. In the following, we focus on a two-dimensional cluster with $L=10$ sites with a periodic boundary condition (Fig. 1)^48^. Results hold qualitatively unchanged for other geometries and sizes as shown below. Optical excitation is induced by electric fields ${\varvec{E}}(t) = - \partial {\varvec{A}}(t)/\partial t$ along the *x*-axis, where the vector potential ${\varvec{A}}(t)$ is switched on as

2 $$\begin{aligned} \begin{aligned} {\varvec{A}}(t)&= A(t) {\hat{x}} = A_0 \cos (\omega _d t) f(t) {\hat{x}}, \\ f(t)&= {\left\{ \begin{array}{ll} \exp \left[ - 4\log (2) \left( \frac{t-\tau _0}{\tau } \right) ^2\right] &{} t < \tau _0 \\ 1 &{} t \ge \tau _0 \end{array}\right. }, \end{aligned} \end{aligned}$$

The form of the excitation is motivated by experiments with ultracold atoms, where driving along one axis is commonly used^26,27^. Our envelop function *f*(*t*) is chosen for computational convenience, while a constant drive amplitude after a linear ramp is used in Refs.^26,27^. We have also checked that fields along the diagonal direction $({\hat{x}}+{\hat{y}})$ bring similar results.

The initial ground state is calculated by the conventional Lanczos method^48–51^, and the subsequent time-evolution is implemented by the Krylov-space method with time step $dt= 0.02$ and Krylov dimension $M = 20$^52–55^. For each time step, we use the midpoint Hamiltonian, while a higher order Magnus expansion is also possible^56^.

To characterize the time-evolved states, we calculate double occupation $d(t) = \sum _i \mathinner {\langle {n_{i\uparrow }n_{i\downarrow }}\rangle }/L$ and kinetic energy $E_\text {kin}(t) = \mathinner {\langle {H_J(t)}\rangle }/L$. For ultracold atoms in an optical lattice, these quantities can be measured by radio-frequency spectroscopy and time-of-flight measurements, respectively^26,27,57^. Since time evolution is unitary, isolated systems do not thermalize as a whole. For a large enough system size, however, local observables do thermalize, as described by the eigenstate-thermalization hypothesis^28^. In driven isolated systems, this leads towards the infinite-temperature state, which is characterized by $d = 0.25$ and $E_{\text {kin}}=0$. We further introduce two quantities to obtain a detailed picture of the time evolution. One is the spectral density of the wavefunction $\mathinner {|{\psi (t)}\rangle }$,

3 $$\begin{aligned} S(t,\omega ) = \sum _{n} |\mathinner {\langle {n|\psi (t)}\rangle }|^2 \delta (\omega -\epsilon _n), \end{aligned}$$

where $\mathinner {|{n}\rangle }$ and $\epsilon _n$ are the eigenstates and eigenenergies (with respect to the ground state) of the zero-field Hamiltonian. Such a spectral decomposition can be approximately calculated by the Lanczos method. The other quantity is effective dimension^39^ or inverse participation ratio^58^,

4 $$\begin{aligned} \kappa (t) = \frac{1}{\sum _n |\mathinner {\langle {n | \psi (t)}\rangle }|^4}, \end{aligned}$$

which quantifies how many of the energy eigenstates are occupied. For example, in the ground state, the spectral density is peaked at zero energy and $\kappa =1$, whereas if the system oscillates between two levels we have $\kappa \approx 2$ and two peaks in the spectral density. The infinite-temperature limit may be defined as the spreading of the wavefunction over all the states of the Hilbert space ${\mathbb {H}}$ and thus $\kappa =\dim {\mathbb {H}}$. We note that Loschmidt amplitudes can be used to calculate the spectral density as well^59,60^.

## Results

**Figure 2:**
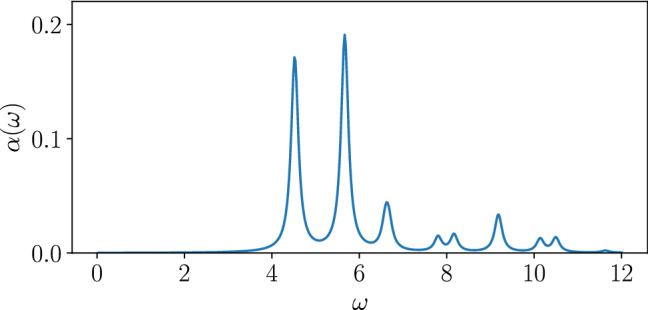
Linear absorption spectrum $\alpha (\omega )$ in Eq. (5) for the two-dimensional case with $L=10$ sites and $U/J_0 = 6$.

Now we turn to the two-dimensional cluster with $L=10$. As a basis to interpret the time-dependent phenomena, let us first look at the linear absorption spectrum^50,61^

5 $$\begin{aligned} \alpha (\omega ) = - \frac{1}{\pi L^2} \mathfrak {I}\mathinner {\langle {0}|} I_x \frac{1}{\omega - H_0 + \epsilon _0} I_x \mathinner {|{0}\rangle }, \end{aligned}$$

where $I_x$ is the total current operator along the *x*-axis, namely along the direction of the optical field [see Eq. (7)]. $H_0$ is the unperturbed zero-field Hamiltonian in Eq. (1). In Fig. 2, we plot $\alpha (\omega )$ for $U/J_0 = 6$ with small broadening factor $\eta = 1/L$. There are three absorption peaks around *U* at $\omega = 4.52, 5.67$, and 6.6, which represent transitions to the one-photon excited states.

### Double occupation

**Figure 3:**
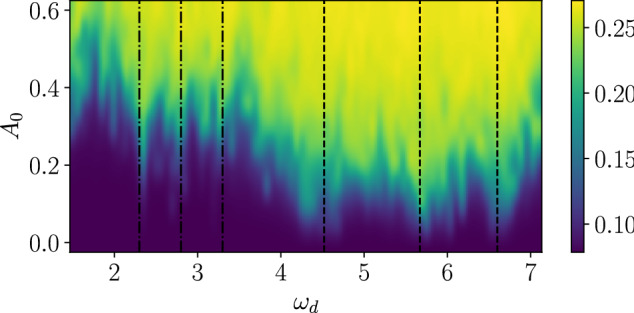
Average double occupation $\overline{d(t)}$ as a function of drive frequency $\omega _d$ and amplitude $A_0$ in the 2D Hubbard cluster ($L=10$). Time average is taken between $t = 30$ and 600. The one- and two-photon resonances are indicated by the dashed and dash-dotted lines, respectively.

**Figure 4:**
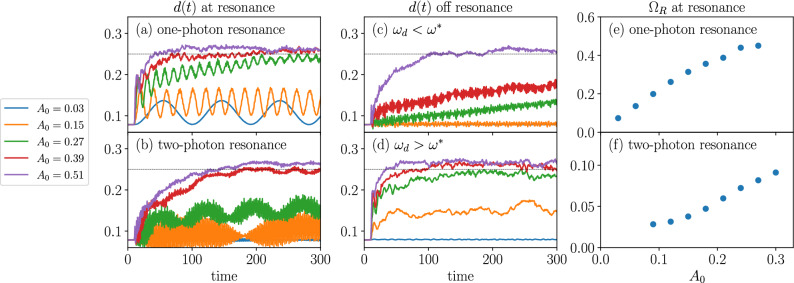
Time evolution of double occupation at the one-photon resonance $\omega _d = 4.52$ (**a**), at the two-photon resonance $\omega _d=2.4$ (**b**), and off resonance (**c**,**d**) in the 2D Hubbard cluster ($L=10$). (**e**,**f**) Amplitude dependence of the Rabi frequency $\Omega _\text {R}$ at resonance.

First, we discuss the average double occupation after the optical fields are turned on. Here the onset of the excitation is set to be $\tau _0 = 10$ with a ramp of width $\tau = 0.1$; we have checked that the steady states are not sensitive to the specific value of $\tau$. This also indicates that the phase of the drive is not decisive for the steady states. The time-averaged values are plotted as a function of drive frequency $\omega _d$ and amplitude $A_0$ in Fig. 3.

As expected, we find strong absorption at frequencies resonant with the three peaks in the optical absorption spectrum $\omega _d\approx 4.52$, 5.67, and 6.63. We notice, however, that even at these resonance conditions, heating is still moderate for small field amplitudes. We find additional two-photon resonances at approximately half the frequencies of one-photon resonances. These resonances stem from second-order processes not captured by the linear absorption spectrum, Eq. (5), and involve states with approximately the same parity under reflection, as we discuss below in more detail. At strong fields, the double occupation saturates to the infinite-temperature value, $d = 0.25$, at all frequencies. The appearance of the infinite-temperature state is in contrast to the two-site model, where pure oscillatory behaviors are observed due to the small dimension of the Hilbert space (see the supplementary material). We also note that there is no resonance at frequencies commensurate to *U*, i.e., $\omega _d = U/n$ with $n \in {\mathbb {N}}$, in contrast to^36^, which uses an infinite dimensional lattice and a strong field amplitude. The discrepancy is probably due to the different lattice geometries or the effect of finite temperatures.

In Fig. 4a we plot, for various field amplitudes, the time evolution of double occupation at the one-photon resonance $\omega _d=4.52$. Similar results are obtained at the other one-photon resonances. Despite the resonance condition, for weak fields, we find FPSs where the double occupation oscillates around a constant value up to $t=2000$ (not shown in the figure). The oscillations can be considered as Rabi oscillations, since their frequencies, $\Omega _\text {R}$, increase approximately linearly with drive amplitude $A_0$ [Fig. 4e]. Below, we explain the linear dependence by time-dependent perturbation theory. Such a FPS with persistent oscillations is one of our main findings. In contrast, for strong fields, the oscillations are on top of an increasing value saturating around the infinite-temperature limit. Kinetic energy approaches to zero in this limit (see the supplementary material). We have confirmed that the same qualitative behavior holds up to $L=14$ sites. As the system size grows, one of the one-photon excited states, which has excitonic nature due to the spin-polaron formation, becomes prominent^62–64^. Thus, we also expect that larger systems show Rabi oscillations by applying a resonant light to this state.

Similar oscillations are found at the two-photon resonance $\omega _d=2.40$ for weak excitation [Fig. 4b]. In contrast to the one-photon resonance, the Rabi frequency depends nonlinearly on the field amplitude [Fig. 4f], because of the two-photon absorption process. We find weak dependence of $\Omega _R$ on $A_0$ at small field amplitudes, which is possibly due to the slight deviation of $\omega _d$ from the resonance. In addition to the slow Rabi oscillations, there exist fast oscillations with frequency $\omega \approx 4.5$, which presumably comes from the coherent superposition of the ground state and the excited states with absorption-peak energies. At large field amplitudes, heating and thermalization to infinite temperature occur.

Away from the resonances, Rabi oscillations disappear and two distinct behaviors arise instead. At weak fields and low frequencies [Fig. 4c], the double occupation increases almost linearly in time. We may consider this linear production of double occupation as an analog of the DC response^65,66^. On the other hand, at weak fields and high frequencies [Fig. 4d], FPSs without Rabi oscillations appear as in^36,41^. Their steady-state values depend on the drive amplitude $A_0$.

As shown in the supplemental material, at resonances, we find monotonic growth of the double occupation and the kinetic energy as the drive amplitude increases. Thus, the total energy also increases monotonically until the system reaches the infinite-temperature state. This amplitude dependence contrasts to a driven two-level system, where absorption saturation occurs for intermediate and large drive amplitudes^67^. The difference comes from the lack of spontaneous emission and the available multi-photon excited states in our model.

### Spectral density

**Figure 5:**
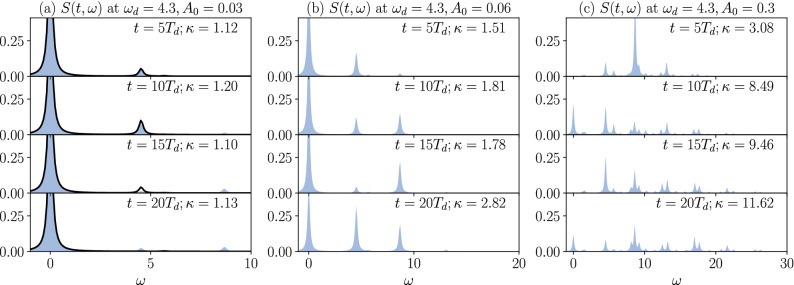
Spectral densities $S(t, \omega )$ for frequency $\omega _d = 4.3$ and amplitude $A_0 = 0.03$ (**a**), $A_0 = 0.06$ (**b**), and $A_0 = 0.3$ (**c**) at times multiples of the drive period $T_d = 2\pi /\omega _d$. The delta functions are approximated by Lorentzian functions. For $A_0 = 0.03$, the approximate expression, Eq. (10), is also plotted (solid line). Effective dimensions $\kappa$ calculated from each spectrum are also shown.

In order to deepen our understanding of the Rabi oscillations and the dependence on the drive frequency and amplitude, we consider the spectral density $S(t, \omega )$ of the time-evolved wavefunction, Eq. (3), and the effective dimension $\kappa$, Eq. (4).

Figure 5 shows the spectral density $S(t, \omega )$ at times multiple of the drive period $T_d=2\pi /\omega _d$ for drive frequency close to the one-photon resonance $\omega _d=4.3$. At small amplitude $A_0=0.03$ [Fig. 5a] the weight oscillates mostly between the ground state and the one-photon excited state. Thus, the effective dimension $\kappa$ remains below 2. During the oscillations, the system repeatedly absorb a photon and release it via stimulated emission^68^. Since our model treats only the classical light fields, spontaneous emission does not occur. Thus, the spontaneous decay of the population in the excited states is not observed. Increasing to $A_0=0.06$ [Fig. 5b], weight on the two-photon excited states $\omega \approx 2 \omega _d$ becomes not negligible, while still the weight is localized on a few states, i.e., $\kappa$ remains below 3. Finally, at strong field $A_0=0.30$ [Fig. 5c], the spectral density spreads over a wide energy range due to multiple-photon absorption and $\kappa > 10$, meaning that the system has reached the infinite-temperature state, i.e., the double occupation and the kinetic energy reaches the infinite-temperature values. We note that due to the limited number of Krylov dimensions (here $M=50$), the effective dimension calculated by the Lanczos method cannot reproduce the bulk infinite-temperature behavior, $\kappa \rightarrow \dim {\mathbb {H}}$.

The spectrum density demonstrates the discrete nature of the multi-photon excited states and gradual filling of these high-energy states with increasing drive amplitudes, which resembles the state-filling effect in semiconductor quantum dots. In the latter, the discrete energy levels are formed by strong spatial confinement of electrons and stronger photoexcitation leads to larger populations in the high-energy levels^69,70^.

### Time-dependent perturbation theory

Here we discuss the physical origin of the Rabi oscillations at the resonant excitation and the amplitude dependence using time-dependent perturbation theory. For this purpose, we define the unperturbed zero-field Hamiltonian $H_0$ and the time-dependent perturbation,

6 $$\begin{aligned} \begin{aligned} V(t)&= H(t) - H_0 \\&= - \sum _{\mathinner {\langle {i,j}\rangle }, \sigma } J_0 \left( e^{-i \frac{e}{\hbar } {\varvec{l}}_{ij} \cdot {\varvec{A}}(t)} - 1\right) c^{\dagger }_{i\sigma }c_{j\sigma } + \text {h.c.}\\&= - I_x \sin [A(t)] - K_x \left\{ 1 - \cos [A(t)]\right\} , \end{aligned} \end{aligned}$$

where $I_x$ and $K_x$ are the current and kinetic-energy operators along the *x*-axis:

7 $$\begin{aligned} I_x = - i\sum _{i, \sigma } J_0 c^{\dagger }_{i\sigma } c_{i-{\hat{x}}, \sigma } + \text {h.c.}, \qquad K_x = - \sum _{i, \sigma } J_0 c^{\dagger }_{i\sigma } c_{i-{\hat{x}}, \sigma } + \text {h.c.}. \end{aligned}$$

Assuming weak perturbation $|A(t)| \ll 1$, we can expand *V*(*t*) as^50^

8 $$\begin{aligned} V(t) \simeq - I_x A(t) - \frac{1}{2} [A(t)]^2 K_x, \end{aligned}$$

which represents the paramagnetic and diamagnetic contributions. Applying first-order time-dependent perturbation theory to the ground state $\mathinner {|{0}\rangle }$, we find the transition amplitude to another eigenstate of the unperturbed Hamiltonian $\mathinner {|{n}\rangle }$ as

9 $$\begin{aligned} \begin{aligned} \mathinner {\langle {n | \psi (t)}\rangle }&\simeq -i \int _0^t \mathinner {\langle {n | V(t) | 0}\rangle } e^{i \epsilon _n t'} dt' \\&\simeq i \mathinner {\langle {n | I_x | 0}\rangle } \int _0^t A(t') e^{i \epsilon _n t'} dt' + \frac{i}{2} \mathinner {\langle {n | K_x | 0}\rangle } \int _0^t [A(t')]^2 e^{i \epsilon _n t'} dt'. \end{aligned} \end{aligned}$$

The first term describes the one-photon absorption process. If the matrix element $\mathinner {\langle {n | I_x | 0}\rangle }$ is peaked at one or a few eigenstates with similar energies, the form has the same structure to the two-level system, and gives Rabi oscillations under continuous excitation $A(t) = A_0 \cos (\omega _d t)$ with $\omega _d \simeq \epsilon _n$^68,71^. From the linear absorption spectrum, Fig. 2a, we see that this matrix element has discrete peaks, which then explains the observed Rabi oscillations. Within the rotating wave approximation, the Rabi frequency is given by ${\Omega _R = \sqrt{(\omega _d - \epsilon _n)^2 + D^2}}$ with $D \propto A_0 \mathinner {\langle {n|I_x|0}\rangle }$. The second term, in addition to the second-order terms, describes two-photon excitation, which gives rise to the nonlinear amplitude dependence $\sim A_0^2$ and to the increase of effective dimension by spreading the weight of the wavefunction to more states [see Fig. 5c]. These terms are responsible for the two-photon resonance at $\omega _d \simeq \epsilon _n/2$.

At the lowest order in *A*(*t*) (i.e., retaining only the paramagnetic term), the spectral density is found to be

10 $$\begin{aligned} S(t, \omega ) \simeq |\mathinner {\langle {0|\psi (t)}\rangle }|^2 \delta (\omega )+ \sum _{n >0} |\mathinner {\langle {n | I_x| 0 }\rangle }|^2 \left| \int _0^t A(t') e^{i \epsilon _{n} t'} dt' \right| ^2\delta (\omega - \epsilon _{n}). \end{aligned}$$

Note that the ground state occupation is calculated as $|\mathinner {\langle {0 | \psi (t)}\rangle }|^2 = 1- \sum _{n>0} |\mathinner {\langle {n|\psi (t)}\rangle }|^2$ to avoid the use of second-order perturbation. In Fig. 5a, the approximate expression, Eq. (10), is compared with the spectral density obtained by the Lanczos method at a weak amplitude, which well reproduces the Rabi oscillations. On the other hand, for resonant excitation, the transition probability becomes too large and makes the perturbative expression invalid. For larger amplitudes, higher orders in perturbation theory are required.

Expressions similar to Eq. (9) can be derived for other types of time-dependent perturbation. An extensively studied example is the driven-interaction protocol^41,58^,

11 $$\begin{aligned} V(t) = \delta U(t) \sum _i n_{i\uparrow } n_{i\downarrow }. \end{aligned}$$

For a weak interaction $J_0/U \gtrsim 1$, the matrix element $\mathinner {\langle {n | n_{i\uparrow } n_{i\downarrow } | 0}\rangle }$ is not peaked nor the excited states are degenerate, and we do not expect Rabi oscillations. However, in the limit of ${J_0/ U \ll 1}$, the excited states are nearly degenerate, and the lower and upper Hubbard bands form an effective two-level system leading to Rabi oscillations^58^.

## Finite-size analysis

**Figure 6:**
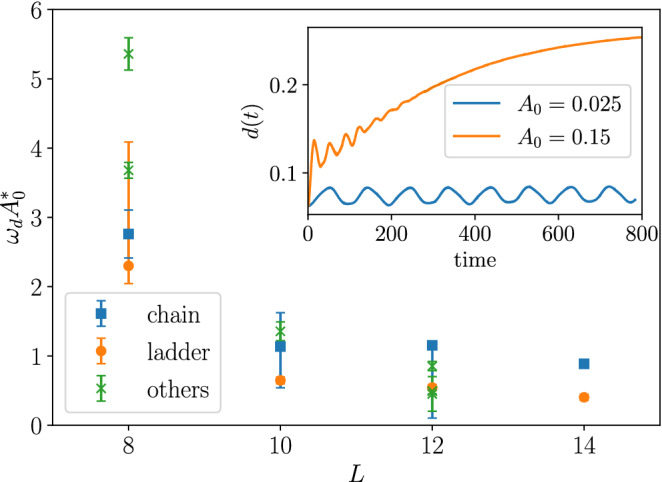
Critical field amplitude $\omega _d A_0^*$ to reach the infinite-temperature state for various lattice geometries. The error bars indicate the regions where the double occupation does not reach a steady state within the simulation time $t \le 1000$. There is no strong size dependence for $L \ge 10$. The inset shows time evolution of double occupation for the $L=14$ ladder.

Up to this point, we have elaborated on a two-dimensional cluster with $L=10$ sites. The essential question is if the Floquet prethermal states that we find for the $L=10$ cluster survive for larger systems. For example, several studies show that the critical drive amplitude to reach the infinite-temperature state vanishes in the thermodynamic limit for different models^38,40^. In order to check the robustness of the results obtained above, here we simulate various sizes of one-dimensional lattices (chains), ladders, and other two-dimensional clusters (see Fig. 1). For each lattice, the drive frequency is taken to be at the lowest one-photon excitation peak in the linear absorption spectrum. In the inset of Fig. 6, we show an example of time evolution of double occupation for the $L=14$ ladder. We confirm that the Rabi oscillations appear for weak drive amplitudes, while the system approaches to the infinite-temperature state for larger amplitudes.

We estimate the critical field amplitudes that separate the prethermal regime $d(t)< 0.25$ and the infinite-temperature regime $d(t) \approx 0.25$, based on the time-averaged double occupation near the end of the simulations $t \sim 1000$. For comparison of different lattices, we convert the drive amplitude $A_0$ to the field amplitude $E_0 = \omega _d A_0$. The obtained critical field amplitudes for various lattices are plotted in Fig. 6. For $L < 10$, due to the limited available absorption processes, the critical field amplitudes are quite large compared to $J_0$. This is consistent with the two-site model (see the supplementary material). For larger systems $L\ge 10$, the critical field amplitudes are of the order of $J_0$. However, there is no systematic decrease as the system size grows as Refs.^38,40^, and thus we expect that the results are valid for larger systems.

## Conclusions

In this work, we have elaborated on the properties of prethermal Floquet states in optically excited Hubbard clusters.We have demonstrated that the prethermal states exist even at resonance, as far as the drive strength is weak. In particular, the Floquet prethermal states at resonance involve Rabi oscillations, whose frequency scales linearly with the field amplitude at one-photon resonances, and with the square amplitude at two-photon resonances. Experimentally, these results can be tested by using ultracold atoms or quantum dot arrays. We have elucidated the origin of the Rabi oscillations by time-dependent perturbation theory. A finite-size analysis has confirmed the robustness of the main results.

The observation of Rabi oscillations in this model suggests possible coherent manipulation of quantum many-body states. Rabi-like oscillations are expected to be a general phenomenon for resonantly driven Hubbard-like clusters; for example, similar oscillations are found after short pulses^72,73^. Considering the coherent nature of Rabi oscillations, the mechanism can be combined with different shapes of drive pulses and used for optical control of correlated quantum states. We expect that introducing dissipation or spontaneous emission^68,74^ to our model will weaken the Rabi oscillations while stabilizing the prethermal Floquet states, and it is an interesting open question to investigate their effects on the final steady states.

## Supplementary information

Supplementary Information 1.
