# Supplementary material for: Floquet prethermalization and Rabi oscillations in optically excited Hubbard clusters
Source: Sci Rep. 2021 Sep 9;11:17994. doi: 10.1038/s41598-021-97104-x (PMC8429774; doi:10.1038/s41598-021-97104-x)
Supplement: Supplementary file 1 — Supplementary Information 1. [file 41598_2021_97104_MOESM1_ESM.pdf]

Supplementary Information: Floquet prethermalization and Rabi oscillations in optically excited Hubbard clusters

Junichi Okamoto[1,2,*] and Francesco Peronaci[3]

[1]Institute of Physics, University of Freiburg, Hermann-Herder-Str. 3, 79104 Freiburg, Germany
[2]EUCOR Centre for Quantum Science and Quantum Computing, University of Freiburg, Hermann-Herder-Str. 3, 79104 Freiburg, Germany
[3]Max Planck Institute for the Physics of Complex Systems, Nöthnitzer Straße 38, 01187 Dresden, Germany
[*]junichi.okamoto@physik.uni-freiburg.de

ABSTRACT

We give a detailed analysis of the two-site model and present results of the kinetic energy.

Two-site model

Here we revisit the two-site Hubbard cluster (see for example Refs.[1,2]) at half-filling under continuous optical excitation in order to illustrate clear Rabi oscillations at frequencies resonant with the system excitation energies and their submultiples. The Hamiltonian for two sites after a local gauge transformation to a purely scalar potential becomes

$$H(t) = -J_0 \sum_\sigma \left(c^\dagger_{2\sigma} c_{1\sigma} + \text{H.c.} \right) + \sum_{i=1}^{2} U n_{i\uparrow} n_{i\downarrow} + \frac{E(t)}{2} (n_2 - n_1). \tag{1}$$

We consider the subspace with particle number $N = 2$ and total spin $S = 0$, which is spanned by the three states

$$|s\rangle = \frac{|\uparrow,\downarrow\rangle - |\downarrow,\uparrow\rangle}{\sqrt{2}}, \qquad |D_\pm\rangle = \frac{|\uparrow\downarrow,0\rangle \pm |0,\downarrow\uparrow\rangle}{\sqrt{2}}. \tag{2}$$

Without the optical field, the Hamiltonian, Eq. (1), is symmetric under the inversion of the two sites. Among the three states, $|s\rangle$ and $|D_+\rangle$ are parity even, while $|D_-\rangle$ is parity odd. This inversion symmetry still holds when the interaction U is time dependent[2], and different parity states are not mixed. In contrast, here the optical field breaks the inversion symmetry and we

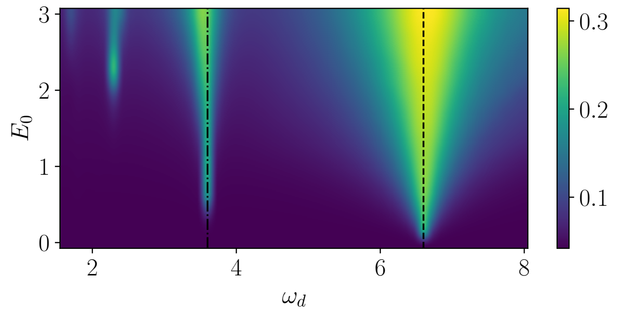

Figure 1. Average double occupation $\overline{d(t)}$ as a function of drive frequency ω_d and amplitude E_0 for the two-site Hubbard model. Time average is taken between $t = 30$ and 600. The one- and two-photon resonances are indicated by the dashed and dash-dotted lines, respectively.

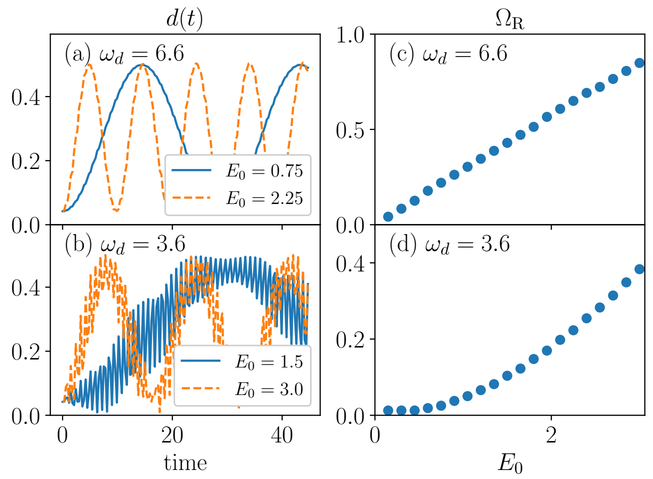

Figure 2. Time evolution of double occupation at the one-photon resonance $\omega_d = 6.6$ (a) and at the two-photon resonance $\omega_d = 3.6$ (b) in the two-site Hubbard model. The amplitude dependence of the Rabi frequency Ω_R is linear (c) or quadratic (d), respectively.

have to consider all the three states; the matrix representation of the Hamiltonian is

$$H(t) = \begin{pmatrix} 0 & -2J_0 & 0 \\ -2J_0 & U & -E(t) \\ 0 & -E(t) & U \end{pmatrix}. \tag{3}$$

At $E(t) = 0$, the ground state $|0\rangle$ and the second excited state $|2\rangle$ are parity even and composed of $|s\rangle$ and $|D_+\rangle$, while the first excited state is parity odd, $|1\rangle = |D_-\rangle$. Their eigenenergies are

$$\varepsilon_{0,2} = \frac{U}{2} \mp \frac{1}{2}\sqrt{U^2 + 16J_0^2}, \quad \varepsilon_1 = U. \tag{4}$$

In the eigenstate basis $\{|0\rangle, |1\rangle, |2\rangle\}$, the Hamiltonian is

$$H(t) = \begin{pmatrix} \varepsilon_0 & -c_1E(t) & 0 \\ -c_1E(t) & \varepsilon_1 & -c_2E(t) \\ 0 & -c_2E(t) & \varepsilon_2 \end{pmatrix}, \tag{5}$$

with $c_1 = \langle 0|D_+\rangle$ and $c_2 = \langle 2|D_+\rangle$. In the Mott insulating regime, $J_0 \ll U$, the nearly degenerate excites states are separated from the ground state by $\sim U$. The structure resembles a cascade type three-level problem and its analytical solutions with Rabi oscillations are found in[1]. It can be seen from Eq. (5) that the optical field only connects the states with different parity. Thus, at first order in the field, starting from the ground state, the only possible transition is $|0\rangle \to |1\rangle$, which is resonant at $\omega_d = \varepsilon_1 - \varepsilon_0$. At second order, on the other hand, we have transitions $|0\rangle \to |1\rangle \to |2\rangle$ resonant at $2\omega_d = \varepsilon_2 - \varepsilon_0$. The former is a one-photon resonance and the latter is a two-photon resonance.

Starting from the unperturbed ground state $|\psi(0)\rangle = |0\rangle$ we calculate its time-evolution under the optical field $E(t) = E_0 \sin \omega_d t$. In Fig. 1, we show the time average of the double occupation $\overline{d(t)}$ as a function of drive frequency ω_d and amplitude E_0. At small amplitudes, large double occupation is observed at the one-photon resonance, $\varepsilon_1 - \varepsilon_0 \approx 6.6$, and at the two-photon resonance, $(\varepsilon_2 - \varepsilon_0)/2 \approx 3.6$.

Figures 2(a) and (b) show the time-evolution of the double occupation at these resonances. We observe nearly perfect oscillations whose frequencies Ω_R depend on the drive strength. In order to show that these stem from the Rabi oscillations, we plot the oscillation frequency Ω_R as a function of E_0 in Figs. 2(c) and (d). At the one-photon resonance, $\omega_d = 6.6$, the oscillation frequency linearly increases with the drive amplitude $\Omega_R \propto E_0$, while at the two-photon resonance, $\omega_d = 3.6$, we find quadratic dependence $\Omega_R \propto E_0^2$. The amplitude dependence resembles that of the Rabi frequency in a two-level system[3], and suggests that the observed oscillations are Rabi oscillations. At the three-photon resonance, $\omega_d \approx 2.3$, oscillating behavior appears only for strong excitation $E_0 \gtrsim 1.0$ and the relation between Ω_R and E_0 is obscure.

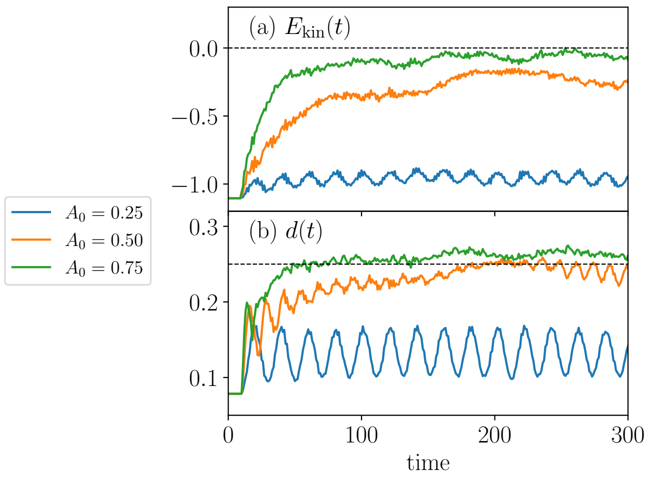

Figure 3. Kinetic energy $E_{\rm kin}(t)$ and double occupation $d(t)$ at the one-photon resonance $\omega_d = 4.52$ in the 2D Hubbard cluster ($L = 10$). Dashed lines denote the infinite-temperature limit, $E_{\rm kin} = 0$ and $d = 0.25$.

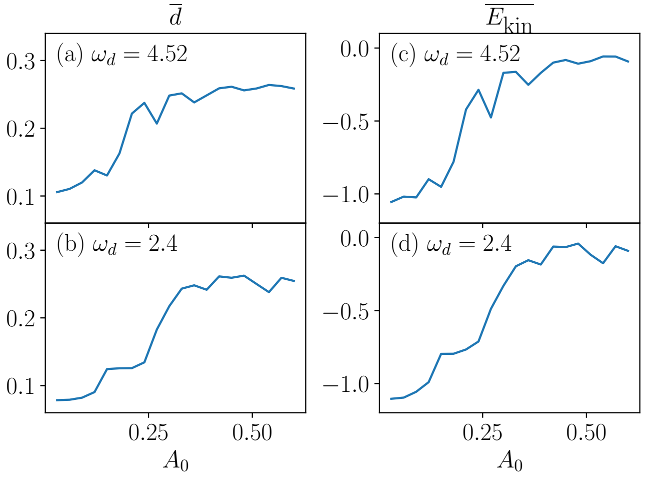

Figure 4. The steady-state values of the double occupation $\overline{d}$ and the kinetic energy $\overline{E_{\rm kin}}$ at at the one- and two-photon resonances $\omega_d = 4.52$ and 2.4 in the 2D Hubbard cluster ($L = 10$).

Kinetic energy

In addition to the double occupation, the kinetic energy also characterizes the infinite-temperature limit, where $E_{\rm kin} \to 0$. In Fig. 3, we plot the time evolution of the kinetic energy at the one-photon resonance $\omega_d = 4.52$ for small and large field amplitudes. For comparison, we also plot the double occupation. The two observables reach steady-state values in similar time scales, which indicates that the absorbed energy is efficiently distributed between the interaction and kinetic energies. At weak excitation, Rabi oscillations appear in the kinetic energy as well, while its relative oscillation amplitude is smaller than that of the double occupation. For strong excitation, the kinetic energy goes to zero, which is consistent with the notion of the infinite-temperature limit.

Figure 4 shows the amplitude dependence of the steady-state values of the double occupation and the kinetic energy at resonances. We see that the steady-state values grow monotonically as the drive amplitude increases. However, the rate of the increase depends on the drive strength nonlinearly. At the one-photon resonance ($\omega_d = 4.52$), the steady-state values grow almost quadratically as A_0 increases and reach the infinite-temperature values, $d = 0.25$ and $E_{\rm kin} = 0$. At the two-photon resonance ($\omega_d = 2.4$), the amplitude dependence of the steady states shows a plateau-like structure around $A_0 \approx 0.2$. In order to fully understand such complex nonlinear amplitude dependence, higher-order calculations, which go beyond the perturbative expression in Eq. (9) in the main text, are necessary.

References

1. Berent, M. & Parzyński, R. Multiphoton resonance in a three-level system with nearly degenerate excited states. *Phys. Rev. A* **82**, 023804, DOI: 10.1103/PhysRevA.82.023804 (2010).
2. Płodzień, M. & Wysokiński, M. M. Rabi-resonant behavior of periodically driven correlated fermion systems. *Phys. Rev. B* **100**, 041116(R), DOI: 10.1103/PhysRevB.100.041116 (2019).
3. Fox, A. M. *Quantum Optics: An Introduction* (Oxford University Press, New York, 2006).
